# Supplementary material for: Are carbon emissions trading and green financial instruments synergistic? -Comprehensive quantitative research based on content analysis
Source: PLoS One. 2024 Mar 7;19(3):e0298601. doi: 10.1371/journal.pone.0298601 (PMC10919601; doi:10.1371/journal.pone.0298601)
Supplement: S1 Data — (ZIP) [file pone.0298601.s001.zip › Data/地级市Z_F工作报告.zip!/╜¡╬≈/╛░╡┬╒≥╩╨/└┤╫╘╣1⁄2╓┌║┼í╛┬φ┐╦╩2╛▌═°í┐_2013.docx]

各位代表：

现在，我代表市人民政府向大会报告工作，请予审议，并请市政协委员和列席的同志提出意见。

2012年工作回顾

去年，在省委、省政府和市委正确领导及你们的监督下，我们团结全市广大干部群众，重点做了以下工作。

一是发展经济。农业克服了低温、早汛和暴雨的影响，实现产值73.3亿元，增长4.5%。粮食总产突破60万吨，提前三年完成“十二五”目标；森林防火、农民安全饮水和农业综合开发工作荣获全省第一名；乐平市和浮梁县分别获得全省新农村建设和清洁工程先进单位。工业克服企业成本上升，资金供给短缺和市场需求减少等困难，实现增加值220亿元，增长14.6%，其中，陶瓷工业增加值63.5亿元，增长15%；航空产业增加值24.9亿元，增长32%。直升机总装园建设已完成前期工作。无人直升机研发中心工程基本完工；江西直升机产业投资管理公司正式运营；焦化集团、昌飞公司改写了景德镇没有百亿企业的历史，产值双双突破100亿元。焦化集团进入中国制造企业500强。华意公司并购西班牙库比克商用压缩机公司，成为全球第二大压缩机生产企业。服务业增加值200亿元，增长19%。陶溪川国际陶瓷文化产业园成为省级服务业集聚区，陶邑文化发展公司成为省级服务业龙头企业；赣东北综合物流园完成“七通一平”；2012瓷博会成功举办，贸易成交总额110亿元；机场年运送旅客量突破40万人次；旅游总收入125.4亿元，增长49.7%。同时，争取重大政策取得突破，资源枯竭型城市转型政策延续五年，成为国家低碳和生态文明试点城市；重大基础设施项目取得新进展，祁浮高速景德镇段建成通车；西瓜洲至中渡口段城防工程按期完工；浯溪口水库移民安置工程和附属设施开工建设；九景衢铁路完成前期工作。

二是扩大开放。引进内资550亿元，增长63%，利用外资1.26亿美元，增长20%；生产型出口5.8亿美元，增长11%；制定了招商项目准入制度，北京等15地招商签约资金415亿元；与长虹集团签订合作协议；与荷、意、韩等国产瓷城市签订了陶瓷发展合作协议，与匈牙利佩奇、意大利法恩扎建立了友好城市；20亿元恒盛化工等201个5000万元以上工业项目签约；全国最大、全省唯一的20兆瓦光伏农业科技大棚等117个项目开工建设；美菱年产200万台冰箱等43个项目竣工投产。

三是深化改革。1、文化体制改革整合了文化管理职能。文艺院团和非时政类报刊转企改制按期完成。2、国有林场改革各项工作全面启动。3、改革城市管理体制，组建了市城市管理行政执法局，环卫管理职能不断向基层延伸。4、改革社会保障体制，覆盖城乡的医疗保险体系基本建立。养老和医疗保险实现全市统筹。5、改革财政体制，实行国库集中支付和部门预算。6、在注册地不变前提下，开展景德镇商业银行战略重组。7、推进服务业综合改革试点。8、推进资源节约型和环境友好型社会建设改革试点。

四是维护稳定。全国“两会”期间非正常赴京访登记为零，确保十八大期间社会稳定；连续11年未发生安全生产重特大事故；保障了食品药品安全；开展了“铁拳”等严打整治专项行动，社会治安态势平稳；圆满完成年度670名征兵任务。妥善处置“1·13”昌汽公司事件，成立维稳工作领导小组，帮助企业恢复生产；正确应对“9·18”涉日保钓游行，引导群众依法理性表达爱国热情；全力抗击“8·10”特大暴雨，在18小时降雨611mm重大灾害前，化解水库和地质灾害险情，转移23.4万人，疏导12趟受阻列车和5900名滞留旅客，实现了“不死一人、不倒一库、不溃一堤”！

五是改善民生。1、城乡低保标准分别由330元和130元提高到360元和170元；2、新农合筹资标准达290元；3、建成廉租房1796套，实物配租1594户，发放廉租补贴1530万元；4、乐平市率先实施城乡一体化供水，为全省树立典范；5、33所中小学被评为省平安校园；6、集中扶持113个市级贫困村的生产开发；7、景德大道等城市主干道实现美化亮化，安装路灯1577盏；8、继2011年后，去年又购买27辆空调公交车；9、老年大学教学楼建设进展顺利；10、方志馆已完成建设。

其它社会事业协调发展。提炼出“大器成景，厚德立镇”的景德镇精神；成为全省唯一的国家版权示范基地；文化部为我市量身定制了文化改革发展政策，创造了文化大发展大繁荣的有利条件；直升机技术主题项目入选国家“863”计划；普通高考录取率高于全省平均水平；乐平市人民医院启动公立医院改革；人口自然增长率控制在7.3‰以内；成功承办全国半程马拉松比赛等赛事。

六是美化城市。新城建设拉开大幕。1、争取到省级低丘缓坡未利用地开发试点政策，规划设计等前期工作基本完成。2、依法依规开展了房屋征收，完成2950亩土地报批手续。3、陶瓷产业发展服务中心和陶瓷总部基地项目列入省重点工程。4、组建了新成建设投资开发公司，确定了融资方案。5、完成水电气等市政设计招标。老城保护全面展开。1、基本完成大遗址保护规划，全市52个点151处窑址完成紫线标识；完成了御窑核心保护区2万㎡房屋搬迁，龙珠阁修缮等重点工程进展顺利；完成了《湖田窑遗址公园规划》，启动湖田窑遗址博物馆立项工作，落马桥古窑遗址保护工程开工建设，完成了唐代南窑和兰田窑全国专家论证。2、完成“一轴四片六厂”工业遗存保护的总体规划、详规及价值评估，宇宙陶瓷创意谷和建国明清窑作群保护工程开工建设。3、老城区保护利用工程完成搬迁2000多户，启动了中华北路天主教堂和神父官厅修缮工程、三闾庙、忠洁侯庙和码头修复工程，95万㎡老城住户搬迁安置房已开工建设。打击“两违”受到国家部委表扬。通过四级联动、属地管理和网格巡查，制止新增“两违”149起，整改到位174起，拆除违章建筑2万多㎡，实现“两违”零增长。

过去一年，我们化解困难，经济社会实现了逆势上扬。预计全年实现生产总值630亿元，增长11.2%；财政总收入84亿元，增长22%；固定资产投资504亿元，增长32%;出口11.8亿美元；单位能耗下降4%；消费品零售总额189亿元，增长14%;城镇居民人均可支配收入21714元，增长14.5%，农民人均纯收入8866元，增长15.5%。

各位代表，成绩归功于党和人民。在此，我代表市人民政府，感谢全市人民的信任和支持！感谢人大和政协的监督！并向所有关心和参与景德镇发展的各界人士表示衷心的感谢和崇高的敬意！

我们清醒地看到，这座陶瓷城市陈旧而苍老的基本状况还没根本转变。经济发展还很困难；社会管理还比较落后；文化资源还未有效保护利用；破坏生态的现象还时有发生；特别是有的政府人员精神懈怠、能力弱、玩心重等问题仍然存在。但我们相信，只要努力，一定会一年更比一年好。

2013年工作安排

按照中央和省里部署，2013年工作总体要求是，全面贯彻落实党的十八大精神，抓改革，扩内需，惠民生，护生态，优先发展陶瓷航空旅游产业，积极融入鄱阳湖生态经济区建设，努力复兴千年名镇，努力让人民过上更加美好的生活。

按照上述要求，今年工作的主要目标是，全市生产总值增长11%；财政总收入增长16%；固定资产投资增长20%；社会消费品零售总额增长15%；实际利用外资增长5%；实际引进内资增长10%；规模以上工业增加值增长14.6%；城镇居民人均可支配收入增长13%；农民人均纯收入增长15%；单位生产总值能耗下降3.6%；居民消费价格涨幅低于3.4%；人口自然增长率低于7.3‰。围绕上述要求和目标，我们将重点做好以下工作。

一、持续健康发展经济

当前，我们面临着经济发展与资源环境约束，扩大就业与产能相对过剩两个矛盾，面临着企业成本上升和创新能力不足的问题。我们应牢牢把握扩大内需这一战略基点，充分发挥消费对经济增长的基础作用和投资对经济增长的关键作用，把经济结构调整作为转变经济发展方式的主攻方向，推进新兴产业，转型升级传统产业，壮大服务业，合理布局基础设施。

（一）夯实基础，发展农业

1、推进农业生产发展。播种粮食139万亩，生产粮食11.8亿斤，市储备粮食0.3亿斤；丰富“菜蓝子”供应，推进茶产业，抓好畜禽养殖，发展林下经济；保护耕地，实现耕地总量动态平衡和占补平衡；抓好病险水库除险和河道整治；建设高标准农田；加强防灾减灾，防控森林火灾和林业有害生物。

2、提升农业产业化水平。建设现代农业示范区；培育现代农业龙头企业，形成龙头企业带动、中小企业、农民专业合作社、科技服务组织竞相发展的新格局；继续推行“企业+合作社+农户”的经营模式；推进农业科技创新；提高农业机械化程度；完善金融保险支持机制；支持农业企业参加各类展示展销活动。

3、实施和谐秀美乡村建设十大工程。1、实施高速公路沿线村庄改造提升工程；2、实施景瑶旅游公路沿线高标准新农村建设；3、以众埠等乡镇为重点，建设村镇联动示范点；4、以洪源镇鸣山村片区为重点，建设村落连片示范点；5、以乐安河沿线70个村庄为重点，建设环境整治和产业调整示范点；6、以湘湖古田村等为重点，建设农村清洁工程示范点；7、以江村严台和勒功沧溪为重点，建设古村保护和旅游发展示范点；8、结合浯溪口库区移民，建设峙滩乡新集镇；9、实施竟成镇和高新区部分城中村新农村建设；10、实施城乡一体化农村安全饮水工程。

4、大力发展县域经济。县（市、区）要发展特色经济。乐平市应在全省保持总量进位、增速领先，争当鄱阳湖生态经济区建设的排头兵；浮梁县应发挥自然资源优势，建设“生态浮梁”；昌江区应服务全市重点项目和航空等高新技术产业，发展现代服务业和休闲生态农业；珠山区应重点壮大服务产业，发展楼宇经济，建设文化和商贸区。

（二）优化结构，发展工业

围绕建设“中国陶瓷特区”目标，按照《加快陶瓷产业发展意见》，重点开展以下工作：1、扶持手工制瓷。手工陶瓷是景德镇人的精神载体，是传承千年的文化遗产。景德镇因手工制瓷而名，因手工制瓷而兴，因手工制瓷而享誉世界！我们一定要积极发掘和扶持隐于市间的良工巧匠，以他们的智慧和劳动，注陶瓷以灵魂和生命，复兴我陶瓷的世界艺术名品和国际奢侈品牌地位！当前要加快建设“手工制瓷名坊园”，帮助15家作坊尽快落户投产；2、振兴日用瓷。以国有企业为主力，全面梳理历代日用瓷精品，按照市场需求、现代时尚、世界潮流，用现代生产技术制造高档日用瓷；3、发展高科技陶瓷。整合部、省、市陶研所和陶院的科研资源，按产学研一体化的要求，加强院所与企业联合，推动高环蜂窝陶瓷等高科技陶瓷产品的发展；4、以确保环境安全和节能减排为前提，合理发展建筑陶瓷；5、加强陶瓷矿产资源保护，实现优质陶瓷原料的有序开采和效益最大化；6、加强陶瓷知识产权保护，尽快颁布实施《景德镇陶瓷知识产权管理规定》和《景德镇瓷器原产地保护标准》，加强 “景德镇” 牌及四大传统名瓷商标管理。

围绕建设航空科技城目标，重点开展以下工作：1、支持昌飞和602所发展。加快建设直升机总装园，三年内形成年产200架直升机产能；加快建设装配制造区“一江两岸”防洪堤坝及排涝工程，建设飞虹大桥；加快建设直升机桨叶等配套项目；支持602所项目研发和产业化，建成南河大桥，建设南河两岸景观带；2、支持和参与江西直升机投资管理公司多渠道筹集资金，加快民用直升机引进和产业化步伐；3、开展直升机产业招商，引进国内外航空制造商及相关配套企业；同时，加强与中央部委及省直有关部门的对接，争取人才资金和项目等方面的支持。

（三）依托优势，壮大服务业

1、提升旅游业水平。强化旅游服务功能，建设旅游服务中心，完善旅游标识系统；挖掘和开发陶瓷文化特色游、自然生态山水游、老城区古镇游、休闲农业乡村游、近郊短线游、森林游等旅游产品；加快景区创建，力争古窑成为5A级景区，高岭·瑶里创建5A级景区；加大旅游促销，提升旅游品牌知名度和美誉度。

2、打造赣东北综合物流园。建设物流批发、仓储、保税区及无水港等十大功能区；重点建设物流园区一期国际商贸物流城、汽车城和大型停车场及配套设施，建设物流园二期路网及市政基础设施，完成罗家滩收费站西迁工作。

3、发展会展经济等其它服务产业。以瓷博会十周年为契机，完善陶瓷会展中心设施，打造国际一流陶瓷博览盛会；提高金融服务水平，完成景德镇商业银行战略重组，支持发展村镇银行等新型农村金融机构，支持服务企业通过股票和债券等方式融资；引进知名品牌和运营商，开展连锁特许经营等新型商贸服务；发展社区服务和家政服务。

在优化产业发展的同时，注重投资对经济发展的关键作用，注重项目和基础设施建设。建设九景衢铁路；建设浯溪口水利枢纽工程；建设瓷都大桥至观音阁城防工程；建设湘官公路和乐安河圩堤。

二、坚定实施开放战略

（一）注重招商引资的重点和实效。陶瓷航空旅游的招商引资应取得新进展，现代服务业、现代农业、文化产业项目引进应取得新突破；有的放矢引进与主导产业相关的大项目，下大气力引进中央企业和知名企业；继续扩大光伏农业科技大棚等项目的进资规模；注重提高招商质量和效益，以招商引资为手段，优化产业结构。

（二）努力做大做强开放平台。1、高新区按照促进跨越发展的《意见》，改革管理体制运行机制，转变职能，提高效率；扩园强区，总面积扩大到39k㎡，并提高园区土地投资强度和产出效益；建设国家新型工业化直升机产业示范基地，推进直升机上下游产品延伸配套，协调发展装备制造、机械家电、新能源、新材料等产业。2、陶瓷园区扩大到20 k㎡以上，引进手工制瓷、高档日用瓷和高技术陶瓷项目，打造一批年税收超千万的陶瓷企业。3、乐平工业园区重点实施产业转型升级，减少企业污染，提高生态环境质量。

（三）密切对外合作交流。扩大与世界产瓷城市的友好交往，通过商贸和特色文化的交流，签订框架协议，结成友好城市，打造经贸合作平台；落实友好城市年度交流合作计划，开展互办“城市日”活动；联系联合国教科文卫组织，全面启动城市整体申遗工作。

三、致力提升城市品位

（一）维护规划权威。1、完善城市规划体系，按照市域和中心城区规划两个层次，完善分区规划，科学编制控制性和修建性详细规划，做好生态、交通、市政和公共设施等专项规划；2、尊重群众诉求，维护公共利益，配置生产要素和各种资源，提高规划科学性、公平性和可行性；3、在完成新一轮城市总体规划修编基础上，将城市基本格局制度化法律化，成为城市建设的总依据。规划是城市建设管理的“宪法”，人人必须始终心存敬畏，绝不允许任何人凌驾于规划之上！绝不允许随意改变规划！绝不允许干扰破坏规划实施！

（二）加快新城建设。1、全面完成新城建设前期工作。完成林地和土地报批，道路和基础设施等专项设计和招投标工作；2、开工建设重点项目。按照统一规划、分步实施、先易后难的策略，优先建设路网、安置房、医院学校、城市规划馆、陶瓷博物馆、档案馆和室内体育馆等重点项目；3、加大融资和招商力度。积极探索多种融资模式，在运用短期融资券或中期票据等融资工具上取得突破；精心谋划项目招商，吸引实力强大的企业参与建设。

（三）严格城市管理。1、理顺市区两级环卫管理体制，市级管理主次干道，并通过公开招标聘请专业企业打扫。区级环卫力量则向城乡结合部、里弄和社区转移。2、切实抓好建筑渣土、生活垃圾和户外广告管理，下决心治理城市“脏”和“乱”。3、下大力气缓解交通拥堵。优化道路布局、公交线路和场站设置，加强出租车和电动车管理，整治非法营运，治理车辆乱停乱放，加快交通基础设施建设。4、巩固打击“两违”工作成果，严防死灰复燃。5、开展市民文明素质提升工程和城市美化专项行动，弘扬景德镇精神。

四、努力保护复兴陶瓷文化

景德镇是文化建筑分布极广的国家文化名城；是传统工业文化遗产极多样的手工业城市；是地下文物极丰富，文化连续性极强的古城。保护千年古镇，传承祖辈遗产，延续陶瓷文脉，就是复兴和运行城市文化，涵养人民精神，创造幸福生活。

（一）保护利用点状分布的古窑业遗址。在52个点151处古窑业遗址中，选择最具代表性窑址，规划建设窑址博物馆。1、御窑遗址保护工程要完成御窑长廊和消防大队等处搬迁，完成龙珠阁等修缮工程，初步展现御窑遗址的美丽轮廓；2、湖田窑遗址保护工程要完成博物馆设计和项目申报，开工建设湖田窑遗址公园; 3、落马桥窑业遗址、唐代南窑遗址、兰田窑和丽阳古窑遗址要深化相关保护工作；4、建设景德镇陶瓷鉴定评估中心和古陶瓷片样本研究馆，保护和利用古陶瓷碎片样本。

（二）保护利用线状分布的现代陶瓷工业遗存。1、全面完成工业遗存核心区及周边地区生态控制性规划，开工建设国际陶瓷雕塑博物馆、景陶大学生创业孵化基地、宇宙艺术创意谷；2、实施建国明清窑作群老坯房和老窑房抢救性修复工程；3、启动陶瓷口述史工程，收集老陶瓷企业历史见证人和技艺传承人的音像和实物资料，打造一个百年陶瓷工业文化展示空间和活着的博物馆。

（三）保护利用片状分布的老街区。按照“留住历史、保护文脉、彰显特色、复兴古镇”的思路，1、继续开展老城区房屋征收补偿和搬迁工作；完善老街区保护利用总体设计方案并实施保护利用工程；梳理区域内的街巷和里弄，保护和恢复历史风貌建筑和非物质遗存，再现原有的街巷体系；2、修复三闾庙码头、古街和忠洁侯庙；3、修缮清代康熙朝天主教堂。我们将努力把老街区打造成山水城合一的城市风光带，展示陶瓷传统的文化集散地，聚集休闲特色的旅游生活区。

（四）增强文化整体实力和竞争力。利用国家文化产业示范基地和国家版权示范基地品牌效应，发展新型文化业态，建立文化产业项目库，扶植一批文化龙头企业；全方位、多形式开展文化推介活动，打造一批富有瓷都特色的文艺精品；培养基层文化骨干、民间文化传承人，组织非遗传承人对外交流，开发更多非遗产品。

五、精心呵护生态环境

景德镇丘陵绵延，水网纵横，森林茂密，空气清新。这是我们最重要的生产力和竞争力，是最宝贵的财富，最大后发优势，保护促进这个优势，既是我们的目标，也是发展的手段。值得警醒的是，我们的生态越来越脆弱；群众对损害生态的容忍度越来越小；生态“未富先衰”的危险越来越大。曾经盛满芙蕖的北沼已难寻踪影。双溪的夜月已被乱搭乱建所遮蔽。市桥的春水也不知去向。挫折和苦头告诉我们：破坏生态的发展，绝不会得到生活的乐趣和发展的希望，只能是生存的艰难！让我们善待这座城市，一心一意，永生不变！

（一）珍爱青山绿水。仁者乐山。我们要敬畏山系，保护自然丘陵，保护山体植被，开展“森林城乡、绿色通道”建设；保护城区的五龙山、凤凰山和马鞍山，遏止推山造地，让绿色山体生活在人间！

水带来生命。昌江和乐安河是养育我们的母亲河，我们要像爱护母亲的眼睛一样守护两河，不能让母亲眼睛流泪！要开展两河流域治理，整治两河污染和非法采砂；建设玉湖国家湿地公园；美化城区的南河、西河和陶溪川；精心呵护浯溪口和共库等饮用水源保护区；完善城市污水管网，实现雨污分流和污水达标排放。

（二）永续利用资源。我们将把国土资源、环境资源和产业发展有效结合起来，实现资源利用效益最大化；支持节能低碳产业和新能源、可再生能源发展；努力实现瓷土、釉矿、紫砂矿和其它非金属矿产资源保护和理性开发；鼓励发展循环经济，促进生产流通消费过程的减量化、再利用和资源化。绝不能让来之不易的资源保护成果被窃取，当成肆意利用的资本！

（三）严禁牺牲环境。尽快编制生态环境保护利用规划，绝不以破坏环境换发展，绝不为眼前利益，牺牲长远发展；严把项目准入关，无论投资额多大，无论效益多好，只要是污染环境的项目，一律不准进入景德镇，违者严肃查处。

（四）保护农村土壤。保护基本农田，保护农产品产地土壤，保护“菜篮子”基地土壤；加强农用土壤环境重点污染源监管。

（五）强化执法监管。充分利用污染源在线监控手段，加强对污染治理设施运转的监察和环境执法，加大主要污染物减排工作力度，严防偷排漏排。

六、切实加强社会建设

社会建设必须以保障和改善民生为重点。我们将着力办好以下实事：1、建成2844套廉租房，2500套公租房，实施1000户农村危房改造；2、提高城乡最低生活保障标准和月人均补差水平，提高农村五保供养标准；3、解决5万农村人口饮水安全问题；4、新建5座垃圾中转站，建设垃圾焚烧处理厂；5、改造茶山路等12条老城区道路，再增加30辆公交车；6、解决城区6个地段积水问题，建设老南河排污集水管道；7、建设20个居家养老服务中心（站），改造提升20个农村敬老院；8、完成童宾路等11个路段的路灯安装；9、建设景北大道等3个地段供水管道；10、免费为生活困难尿毒症患者血透，免费为符合条件的心脏病儿童治疗，免费为六类城市居民提供总额为360万元基本公共卫生服务。

同时，新增10万个就业岗位；推进社会保障的扩面征缴；完善社会救助体系；办好人民满意教育，构建终身教育体系；建设国家创新型试点城市；建设“智慧景德镇”信息化门户；改革基层医疗卫生机构；监管食品药品安全；抓好人口和计划生育；改进民兵工作，提高民兵预备役建设质量；举办全市第十二届运动会暨首届全民健身运动会；提升市民法制意识；抓好安全生产；建设社会管理信息平台，完善社会风险评估机制和治安防控体系，增强人民群众安全感。

各位代表，我们正走在迈向“小康生活”的大道上。未来的景德镇人民还要过上“精致生活”，过上“艺术生活”。这样美好幸福的生活，需要每个人奉献心血和汗水，政府要负责，群众要参与，需要每个人抛弃私心私利，服从国家和公众利益；别问这座城市能为我们做什么，而是问问自己能为这座城市做什么！

让我们高举中国特色社会主义伟大旗帜，在市委领导下，凝心聚力，共同绽放生命的光彩，让这座城市更有尊严！让城市的人民更有尊严！让人民的生活更有尊严！
